# A Válvula Esquecida não deve ser Abandonada: Impacto da Insuficiência Tricúspide nos Resultaods Clínicos após Implante de Válvula Aórtica Transcateter

**DOI:** 10.36660/abc.20230410

**Published:** 2023-07-21

**Authors:** Gabriel Kanhouche, Henrique B. Ribeiro

**Affiliations:** 1 Hospital das Clínicas Faculdade de Medicina Universidade de São Paulo São Paulo SP Brasil Instituto do Coração do Hospital das Clínicas da Faculdade de Medicina da Universidade de São Paulo, São Paulo, SP – Brasil

**Keywords:** Estenose Aórtica, Regurgitação Tricúspide, Insuficiência Tricúspide, Dano Cardíaco Extravalvar, Implante Transcateter de Válvula Aórtica, TAVI

A estenose aórtica (EAo) grave é uma doença frequente e potencialmente grave se não for tratada. Apesar da ampla adoção da substituição cirúrgica da válvula aórtica (SAVR) e, mais recentemente, do implante transcateter da válvula aórtica (TAVI), as taxas de mortalidade no seguimento de médio e longo prazo permanecem relativamente altas, especialmente para pacientes de mais alto risco.^1 , 2^ Da mesma forma, embora a TAVI transfemoral tenha se tornado a abordagem dominante para pacientes com idade > 70 anos, permitindo uma redução significativa nas taxas de mortalidade por todas as causas e cardíacas em comparação à SAVR, o prognóstico de determinados subgrupos de pacientes ainda está comprometido.^3^ Portanto, estudos anteriores na área de TAVI têm se concentrado em ajudar na estratificação de risco desses pacientes. Por exemplo, Généreux et al.^3 , 4^ demonstraram que a extensão do dano cardíaco entre pacientes com EAo submetidos ao TAVI pode desempenhar um papel importante. Assim, um impacto gradual em mortalidade no seguimento foi demonstrado quando os pacientes com EAo também apresentavam comprometimento da câmara esquerda, com ou sem regurgitação mitral, seguida de hipertensão pulmonar, com ou sem insuficiência tricúspide (IT) e, finalmente, com disfunção do ventrículo direito.^4^ Vale ressaltar que os dois últimos estágios (com IT moderada ou grave e disfunção do ventrículo direito) representam a maior taxa de mortalidade, chegando a 20% em 1 ano.^4^ Além do grau de IT, há controvérsia sobre se as mudanças no grau IT no seguimento também podem desempenhar um papel nos resultados clínicos.

Nesta edição do jornal, Erbano et al.^5^ avaliaram a associação entre IT e mortalidade em uma revisão sistemática e metanálise de pacientes com EAo submetidos ao TAVI, tendo também avaliado as mudanças na gravidade da IT pós-TAVI e sua relação com a mortalidade a curto e médio prazos. O desfecho primário foi definido como a incidência de mortalidade por todas as causas de acordo com os graus de IT pré-procedimento. Os desfechos secundários incluíram mortalidade cardíaca e hospitalização por insuficiência cardíaca. Os autores incluíram 24 estudos, com mais de 45.000 pacientes, cuja idade média foi de 81,7±8,5 anos; 52% eram do sexo feminino, com média de escore de risco pelo Society of Thoracic Surgeons (STS) de 8,2±6,0. Aproximadamente 22% dos pacientes apresentavam IT moderada ou grave no início do estudo. Após um acompanhamento médio de 1,2 anos, a análise agrupada de 14 estudos revelou que graus mais altos de IT foram associados a um pior prognóstico. A IT grave foi associada a um aumento de 83% na mortalidade em comparação com IT leve no curto prazo e 56% no médio prazo. Na análise agrupada, a persistência de graus moderados/graves de IT, após um seguimento médio de 21 meses pós-TAVI, também foi associada a um aumento de 2 vezes na mortalidade por todas as causas.

Estudos prévios no campo TAVI ressaltaram o potencial impacto do dano cardíaco nos resultados clínicos em pacientes com EAo submetidos a SAVR e TAVI.^5 , 6^ Nesses estudos, a gravidade da IT e o envolvimento do ventrículo direito foram fatores-chave que determinaram maior mortalidade (~2,2 vezes em 2 anos de seguimento em TAVI e 1,6 vezes em 2 anos de seguimento em SAVR).^6 , 7^ O presente estudo amplia ainda mais essas observações, demonstrando um impacto nos desfechos clínicos de curto e médio prazo de acordo com a gravidade da IT após TAVI, mesmo após múltiplos ajustes para fatores de confusão. A EAo grave causa sobrecarga de pressão resultando em remodelamento cardíaco do ventrículo esquerdo, seguido de envolvimento das câmaras cardíacas direitas em estágios avançados.^8 , 9^ Se a IT representa apenas um marcador substituto de uma doença mais avançada ou um fator de risco, ainda permanece incerto.

Outro ponto importante nesta metanálise é que a gravidade da IT melhorou em pelo menos um grau em ~40% dos pacientes. Pacientes sem melhora no grau da IT pós-procedimento apresentaram piores resultados e qualidade de vida.^5^ Em outro estudo, Genereux et al. também demonstraram que a progressão ou regressão do dano cardíaco extravalvar no seguimento de 1 ano pós-TAVI pode se associar ao prognóstico.^3^ Pacientes com piora do dano cardíaco após 1 ano tiveram 95% mais chance de mortalidade.^7^ O impacto significativo da gravidade da IT nos desfechos clínicos pode estar relacionado a fatores clínicos como fibrilação atrial, hipertensão pulmonar e disfunção do ventrículo direito. Por exemplo, Granot et al. demonstraram mortalidade 3,3 vezes maior após TAVI entre pacientes com gravidade de IT persistente e disfunção do ventrículo direito.^2 , 10^

Em conjunto, esses achados sugerem que o manejo precoce da EAo, antes que ocorra dano cardíaco extravalvar, pode melhorar o prognóstico e reduzir a mortalidade. De fato, mesmo no tratamento de pacientes com EAo grave assintomática, realizar intervenção precoce com TAVI independentemente da condição cardíaca (EARLY TAVR; ClinicalTrials.gov: NCT03042104) ou entre aqueles com sinais incipientes de descompensação do ventrículo esquerdo, apesar da ausência de sintomas (EVOLVED; ClinicalTrials.gov: NCT03094143), também poderão mostrar um impacto significativo nos resultados clínicos em um futuro próximo. Enquanto isso, entre pacientes com doença mais avançada e maior gravidade da IT, a combinação de múltiplas intervenções transcateter tanto na valva aórtica quanto em outras valvas com doença concomitante significativa (ou seja, mitral e tricúspide) também podem melhorar os resultados clínicos.^11^ A presente meta-análise nesta edição, certamente esclareceu a importância da gravidade da IT em pacientes submetidos ao TAVI. Enfatiza a importância de um diagnóstico imediato e um plano de tratamento bem elaborado para pacientes com EAo, incluindo várias especialidades, tratamento clínico otimizado e intervenções adequadas e oportunas para melhorar os resultados clínicos em um grupo tão complexo e heterogêneo de pacientes.
