# Supplementary material for: A Válvula Esquecida não deve ser Abandonada: Impacto da Insuficiência Tricúspide nos Resultaods Clínicos após Implante de Válvula Aórtica Transcateter
Source: Arq Bras Cardiol. 2023 Jul 21;120(7):e20230410. [Article in Portuguese] doi: 10.36660/abc.20230410 (PMC10421601; doi:10.36660/abc.20230410)
Supplement: Translation

# Translation: The Forgotten Valve is not to be Forgiven: Tricuspid Regurgitation Impact on Clinical Outcomes after Transcatheter Aortic Valve Implantation

**Authors:** Gabriel Kanhouche, Henrique B. Ribeiro

**Affiliations:** 1 Brasil Instituto do Coração do Hospital das Clínicas da Faculdade de Medicina da Universidade de São Paulo, São Paulo, SP – Brasil

**Keywords:** Aortic Stenosis, Tricuspid Regurgitation, Extravalvular Cardiac Damage, Transcatheter Aortic Valve Implantation, TAVI

Severe aortic stenosis (AS) is a frequent and potentially severe disease if left untreated. Despite the widespread adoption of surgical aortic valve replacement (SAVR) and more recently transcatheter aortic valve implantation (TAVI), mortality rates in the mid- to long-term follow-up remain relatively high, especially for higher-risk patients.^1 , 2^ Yet, transfemoral TAVI became the dominant approach for patients with age > 70 years, enabling a significant reduction in the all-cause and cardiac mortality rates with respect to SAVR, even though the prognosis of a certain subgroup of patients is still jeopardized.^3^ Therefore, prior studies in the TAVI field have focused on helping in risk stratifying such patients. For instance, Genereux et al.^3 , 4^ have shown that the extent of cardiac damage among patients with AS undergoing TAVI could play an important role. Accordingly, a stepwise impact in the mortality at follow-up was demonstrated when patients with AS also had left chamber involvement, with or without mitral regurgitation, followed by pulmonary hypertension, with or without tricuspid regurgitation (TR), and finally with right ventricle dysfunction.^4^ Of note, the last two stages (with moderate or severe TR and right ventricle dysfunction) represent the highest mortality rate, up to 20% at 1 year.^4^ Apart from TR grade, there is controversy on whether the changes in TR grade in the follow-up might also affect the clinical outcomes.

In this journal issue, Erbano et al.^5^ evaluated the association between TR and mortality in a systematic review and meta-analysis of patients with AS undergoing TAVI and further assessed the changes in TR severity post-TAVI and its relationship with short and mid-term mortality. The primary endpoint was defined as the incidence of all-cause mortality according to TR grade at baseline. Secondary endpoints included cardiac mortality and heart failure hospitalization. The authors included 24 studies and more than 45.000 patients, whose mean age was 81.7±8.5 years; 52% were female, with a mean Society of Thoracic Surgeons (STS) score of 8.2±6.0. Approximately 22% of patients had moderate or severe TR at baseline. After a mean follow-up of 1.2 years, pooled analysis of 14 studies revealed that higher grades of TR were associated with a worse prognosis. Severe TR was associated with an 83% increased mortality compared to no/mild TR in the short-term and 56% in the mid-term. In the pooled analysis, the persistence of moderate/severe TR grades, after a mean follow-up of 21 months post-TAVI, was also associated with a 2-fold increase in all-cause mortality.

Prior studies in the TAVI field have underlined the potential impact of cardiac damage on clinical outcomes among patients with AS undergoing SAVR and TAVI.^5 , 6^ In such studies, TR severity and right ventricle involvement were key factors determining greater mortality (~2.2-fold at 2 years follow-up in TAVI and 1.6-fold at 2 years follow-up for SAVR).^6 , 7^ The present study extends these observations further, demonstrating both an impact in the short- and mid-term clinical outcomes according to TR severity after TAVI, even after multiple adjustments for confounders. Severe AS causes pressure overload resulting in cardiac remodeling of the left ventricle, followed by right cardiac chambers involvement in an advanced stage.^8 , 9^ Whether TR represents only a surrogate marker of a more advanced disease or a risk factor remains uncertain.

Another important point in this meta-analysis is that TR severity improved by at least one grade in ~40% of patients. Patients without improved TR severity post-procedure presented worse outcomes and quality of life.^5^ In another study, Genereux et al. also demonstrated that progression or regression of extravalvular cardiac damage after 1 year post-TAVI may associate with prognosis.^3^ Patients with worsening cardiac damage after 1 year had a 95% more chance of mortality.^7^ The significant impact of TR severity on clinical outcomes might be related to clinical factors such as atrial fibrillation, pulmonary hypertension, and right ventricle dysfunction. For instance, Granot *et al.* demonstrated 3.3-fold increased mortality after TAVI among patients with persistent TR severity and right ventricle dysfunction.^2 , 10^

Collectively, these findings suggest that earlier management of AS, before extravalvular cardiac damage ensues, may improve prognosis and reduce mortality. Indeed, even in the treatment of patients with asymptomatic severe AS, performing an earlier intervention with TAVI irrespective of the cardiac condition (EARLY TAVR; ClinicalTrials.gov: NCT03042104) or among those with incipient signs of left ventricle decompensation, despite the absence of symptoms (EVOLVED trial; ClinicalTrials.gov: NCT03094143), might also show a noteworthy impact on the clinical outcomes in the near future. Meanwhile, among patients with more advanced disease and TR severity, the combination of multiple transcatheter interventions both in the aortic valve and other concomitant valve diseases (i.e., mitral and tricuspid) might also enhance clinical outcomes.^11^ The present meta-analysis in this issue has certainly shed some light on the importance of TR severity among patients undergoing TAVI. It emphasizes the importance of a prompt diagnostic and a well-elaborated treatment plan for patients with AS, including multiple specialties, optimal medical treatment, and proper, timely interventions to improve clinical outcomes in such a complex and heterogeneous group of patients.
